# Influence of ovarian hormones on occurrence of cortical spreading depression and its suppression of by L-kynurenine in rat

**DOI:** 10.1186/1129-2377-14-S1-P84

**Published:** 2013-02-21

**Authors:** V Chauvel, S Multon, J Schoenen

**Affiliations:** 1 GIGA Neurosciences, Belgium

## Background

Migraine is sexually dimorphic and associated in 20% of patients with an aura of which cortical spreading depression (CSD) is the most likely cause. We have previously shown that CSD is suppressed by L-kynurenine (L-KYN), the precursor of kynurenic acid and that this effect depends on the stage of the estrous cycle in female rats [1].

## Objective

To determine the influence of ovarian hormones on the suppression of KCl-induced CSD by L-KYN by directly modulating estradiol or progesterone levels in ovariectomized rats.

## Methods

Four groups of adult female rats (n = 16) were ovariectomized and subcutaneously implanted with silastic capsules filled either with progesterone or 17β-estradiol mixed with cholesterol (groups 1 & 2), with cholesterol only (group 3) or left empty (group 4). Two weeks after the ovariectomy/capsule implantation, the animals received an i.p. injection of L-KYN (300 mg/kg) or NaCl as control. Thirty minutes later CSDs were elicited by applying 1M KCl over the occipital cortex and recorded by DC electrocorticogram for 1 hour.

## Results

Both 17β-estradiol and progesterone significantly enhance CSD frequency. Progesterone levels significantly influence the L-KYN effect on CSD frequency (interaction treatment (L-KYN vs NaCl) x Prog. level (high vs low)), L-KYN being more efficient to reduce CSD occurrence when the progesterone level is high. By contrast, 17β-estradiol levels do no significantly influence the suppressive effect of L-KYN on CSD.

## Conclusion

Our findings show that both estrogens and, more surprisingly, progesterone increase CSD susceptibility. This might be relevant for the aggravation/appearance of migraine with aura during pregnancy or intake of a combined contraceptive pill. Furthermore, L-kynurenine is more efficient in suppressing CSD when levels of progesterone are high. Taken together, these results emphasize the complex role of sex hormones in migraine and may open novel perspectives for its preventive treatment [2].
